# Result-Scalability: Following the Evolution of Selected Social Impact of HPC

**DOI:** 10.1177/10943420251338168

**Published:** 2025-04-24

**Authors:** Sally Ellingson, Guillaume Pallez

**Affiliations:** 1 University of Kentucky; 2 Inria

**Keywords:** HPC, social impact, scalability, top500

## Abstract

While the scientific community traditionally relies on various computational metrics to assess the performance of HPC systems –such as the TOP500 list (based on HPL performance), HPCG, Graph500, IO500– these metrics do not capture how HPC contributes to social progress. We propose a novel approach to follow how the growth of HPC systems and the advances of HPC research address concrete social challenges. The uniqueness of these new metrics lies in their ability to not only measure the capabilities of HPC architectures but also to gauge the concrete social advancements achieved through their use: it focuses on the output of the computation instead of its input. Contrarily to current measure, it also promotes the diversity of machines by evaluating the Pareto front created between size and result. We emphasize the need for dynamic, community-driven metrics that can evolve with emerging social needs.

## Introduction

High-Performance Computing is expensive. The largest machines currently cost hundreds of millions of dollars to build; their power consumption and ecological impact is that of a city of tens of thousands inhabitants. Yet they are fundamental cornerstones of science, and they are used to run many applications with large social impact.

To motivate the construction of new supercomputers, HPC centers often describe the applications that are going to use them, and why we need such scale Committee on Physical and Sciences (1992); Genci (2021). Amongst the core applications, HPC is often described as an enabler of better climate understanding, a tool that allows rapid prototyping and advanced manufacturing techniques, particularly in fields that require flow modeling such as avionics. It is also described as an important strategic tool for national security and new energy development. As part of the Cancer Moonshot, the Department of Energy and the National Cancer Institute forged a collaboration to accelerate precision oncology with scientific computing at scale. Projects range from understanding the underlying biological mechanisms of certain cancers, to cancer surveillance and methods to enhance the discovery of new cancer therapies. The progress made on these various fields are highlighted each year at the SC’XY conference* with the attribution of the ACM Gordon Bell prize^†^. These works describe new scientific discovery that was made possible through the use of supercomputers. In these various presentations, our community shows that we use these machines for new scientific discoveries.

### On the importance of scale

In these description, the demonstration of the need for scale is often provided through an evaluation of the size of the input that can be analyzed, or the time to solution. Rarely the output of the computation is analyzed. In computing terms, we tend to talk about **weak scaling** –how the solution time varies with the size of the platform when the problem size grows with the platform size–, or **strong scaling** –how the solution time varies with the size of the platform for a fixed total problem size. Yet, we rarely demonstrate the importance of scale from the solution perspective: how the quality of the solution evolves with the size of the platform, a **result scaling** metric. Specifically, at this time and contrarily to weak/strong scalability demonstration, result-scalability is not a requirement when performing a compute request in many of the large scale centers Felker et al. (2024).

### The motivation for Result-scalability

While designed for the scientific fields of human experimentation, the Nuremberg code^‡^ (1947) reminds us of an important ethical statement for research:

The experiment should be such as to yield fruitful results for the good of society, unprocurable by other methods or means of study, and not random and unnecessary in nature.

It stresses how scientific research should be designed to benefit society, but also that the methods should not be unnecessary in nature. This is repeated in many ethical codes such as the ACM code of ethics (“A computing professional should contribute to society and to human well-being”). Going one step further, as scientists, it is our responsibility to demonstrate to the general public and to policy-makers that what we are working on benefits society without unnecessary harm.

The Dougnut theory Raworth (2012) has discussed how in a limited world, the role of progress is to find the sweet spot between social benefits and the ecological planetary limits. Yet we cannot do this if we do not understand the relation between a computation and the social benefit it brings. Currently, our ways to measure the advances allowed by High-Performance Computing are very computing-oriented.

On the one hand, some metrics demonstrate how HPC is able to increase the performance of new machines through specific benchmarks –HPL Luszczek et al. (2005), HPCG Heroux et al. (2013), Graph500 Murphy et al. (2010); IO500 Kunkel et al. (2016)–. The relation between these metrics and social advances is implicit, but never really demonstrated: it is assumed that the more performance the better we can answer social questions.

Fundamentally, these metrics measure the amount of hardware an HPC center needs to purchase in order to competitively run the various benchmarks, but they do not really measure how increasing the size of HPC machines benefits society.

On the other hand, application domains study how they can improve the precision of their simulations through the use of a larger compute resource. Again, the implicit assumption is that more precision can only help to solve more social problems. Yet this is not generally true. One of the main examples of this is the use of mixed precision Baboulin et al. (2009) for scientific computing, where sometimes, the loss in precision used during the computation does not reflect in a global loss in the final result. It has been pushed to the edge in deep learning Gupta et al. (2015) where int8 precision is used whenever possible.

**Figure 1.**
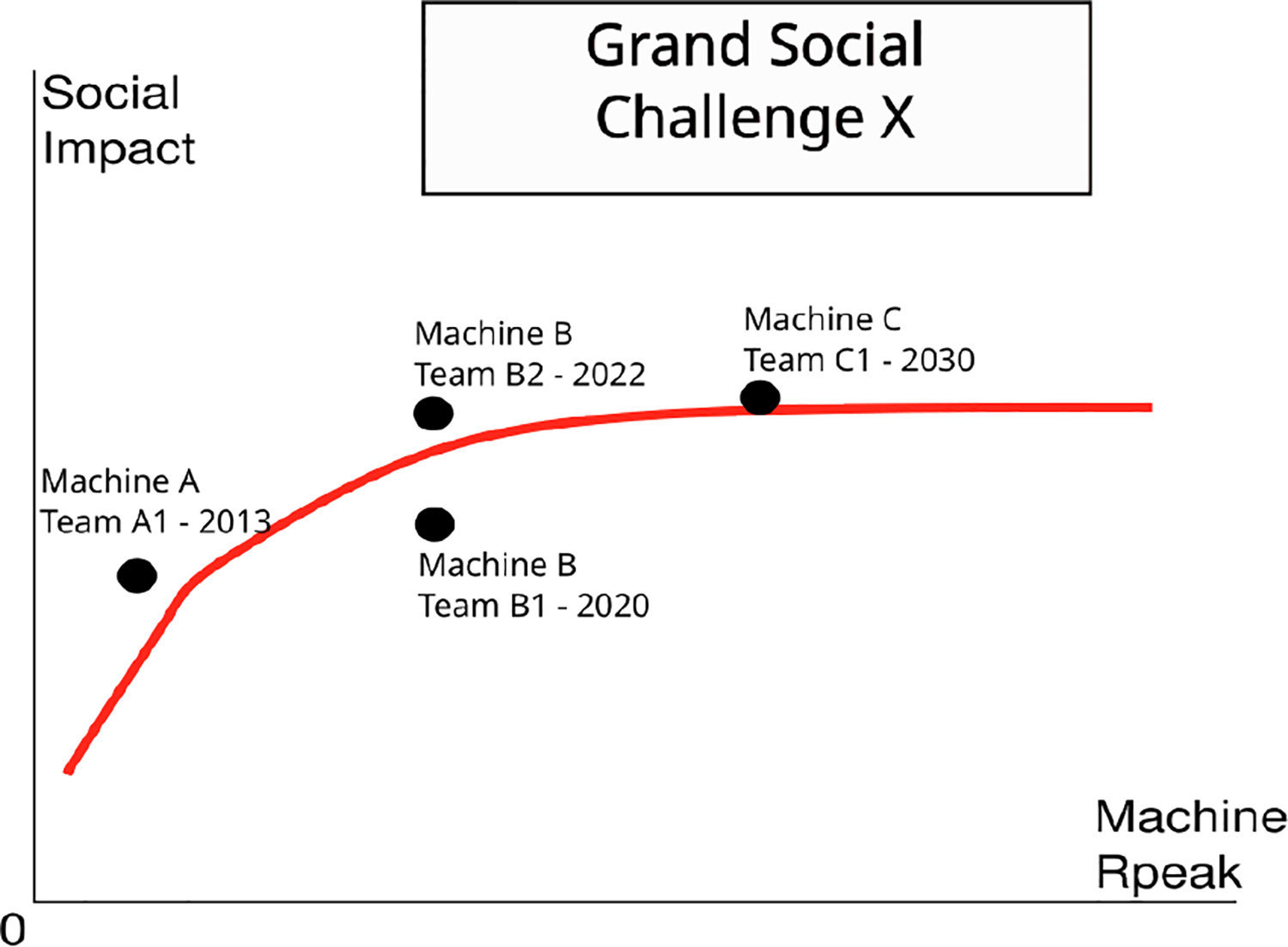
Being able to measure the social advance as a function of scale would help not only to showcase how architecture design helps resolve social problem, but also how algorithmic advances allow it. It also showcases the trade-off between scale and impact.

Here we propose a way to measure some of the social advances that are possible thanks to system put at scale through the use of an evaluation of Grand Social Challenges (see Figure 1). We discuss the limits and complexity of proposing such metrics, and how one can try to solve them.

## Grand Social Challenges

**Figure 2.**
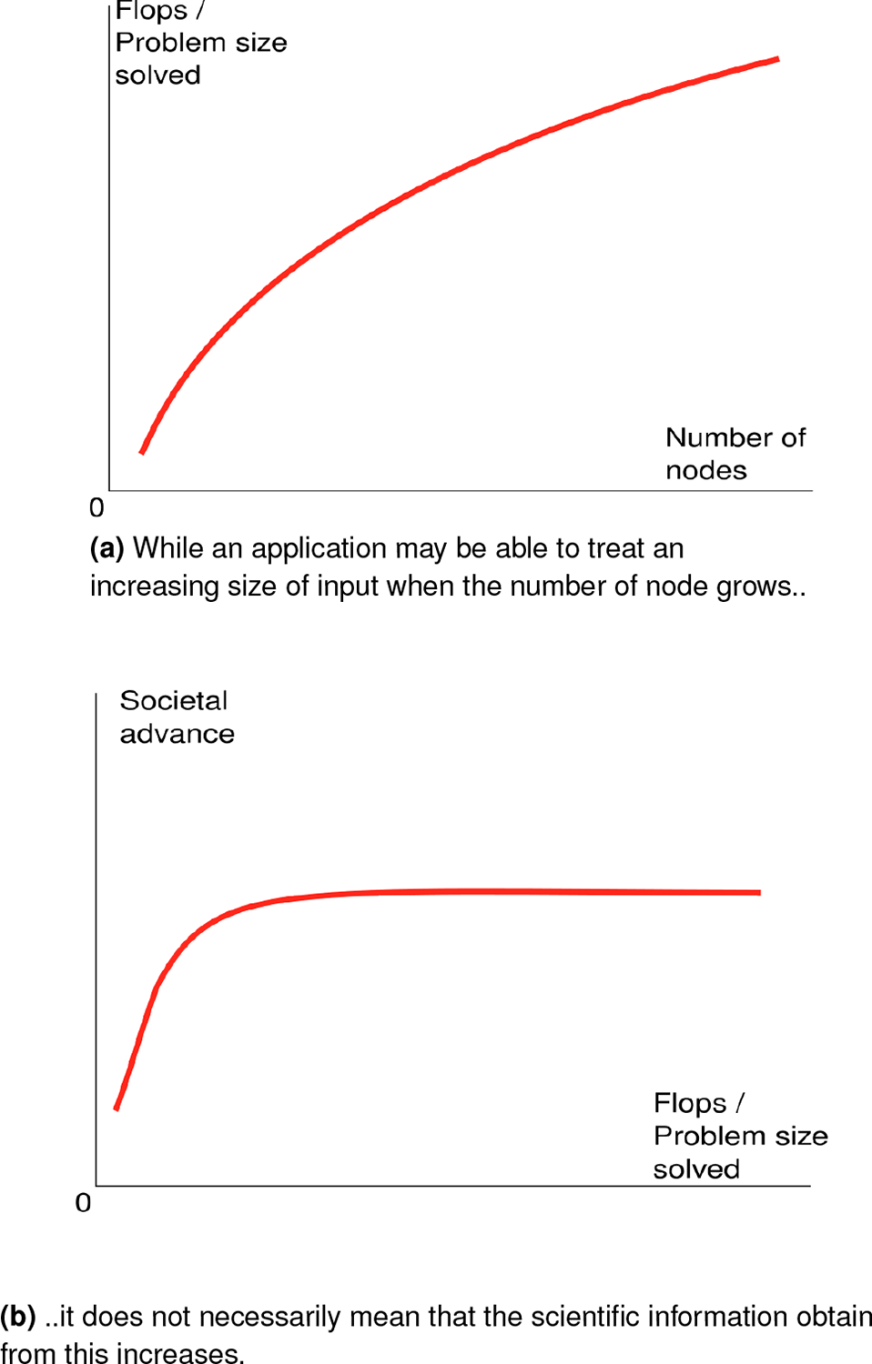
There is a difference between how well a parallel function scales, and the information that one can extract from this performance.

While benchmark-based metrics are of tremendous importance for computer scientists interested to understand the various bottlenecks in HPC machines, from a policy-maker or tax-paying citizen perspective, these evaluations are not entirely helpful. Indeed, they do not justify **why, from a social perspective, we need increasingly larger machines**, and why our government should invest in HPC. There is no natural linear correlation between the ability of a problem to scale, and the social advance this scale brings (see Fig. 2).

### The limits of Weak/Strong Scaling

Focusing on weak and strong scaling brings obvious benefits, particularly when Technical Committees evaluate the ability for an application to run on a supercomputer. Yet their expressiveness also has obvious limits. They are application dependent and can only be used to compare the scalability of a single solution. They miss the opportunity to follow how algorithmic advances allow us to impact society. In the extreme case, one could imagine two solutions for the same societal problem:

- One relying on a with extremely high computing intensity, focusing on a very precise model that scales extremely well;
- The other would choose another approach, less compute intensive, highly optimized and hence with poorer scalabilty performance, although able to obtain the same social results.

By simply looking at weak/strong scalability, the first solution could seem like the best choice. However, by looking at result scaling, one would determine the second choice to be the best because the same social results are obtained with less resources. The goal of this proposal is to highlight this alternative by focusing on *result-scalability*.

In this section, we first provide an informal definition of what a social challenge is. Then we discuss the difficulties in designing such grand challenges along with recommandations to solve these limits. We detail what a challenge submission would look like based on these recommandations. Finally we discuss the limits of such challenges, and the risks with the creation of such challenges.

### What do we call a Grand Social Challenge?

Here we propose to study social questions based on HPC applications, where the impact of scale can be measured in concrete terms. Note that the frontier between a question that solves a scientific problem, and a question that solves a social question is tenuous.

A social challenge should be an answer to an issue. It answers an expressed need by society.

*Climate Models* To provide an example, consider a farmer that needs to plan its harvest over a period of time. They want to know with some lead time and some accuracy the weather.

1. *What is the best resolution/number of grid point that I can use when simulating a climate model as a function of the number of Flops of my machine?* is a scientific question: it is an intermediate step to get to the solution of the social question.
2. *What is the lead time for a climate prediction with 80% accuracy as a function of the number of Flops of my machine?* is a social question: it answers an expressed social need. It is often the output of the computation that was performed.
3. *Given the cost of Flops, and the information I would gain, should I run this experiment at this scale?* is a utility question, based on economical and/or political considerations. It focuses on how one uses the result to the social question.

#### Social Challenge evaluation

Some domain-scientists have started to measure how their fields have evolved through time. This is for instance the case of the European Centre for Medium-Range Weather Forecasts (ECMWF). Among others, they are following how their models evolve throughout time, through the use of the Anomaly Correlation Coefficient (ACC). As described on the ECMWF wiki ECMWF (2021): *ACC is a measure of how well the forecast anomalies have represented the observed anomalies. It shows how well the predicted values from a forecast model “fit” with the real-life data. ACC values lie between +1 and −1. Where ACC values:*

- approach +1 there is good agreement and the forecast anomaly has had value.
- lie around 0 there is poor agreement and the forecast has had no value.
- approach −1 the agreement is in anti-phase and the forecast has been very misleading.

**Figure 3.**
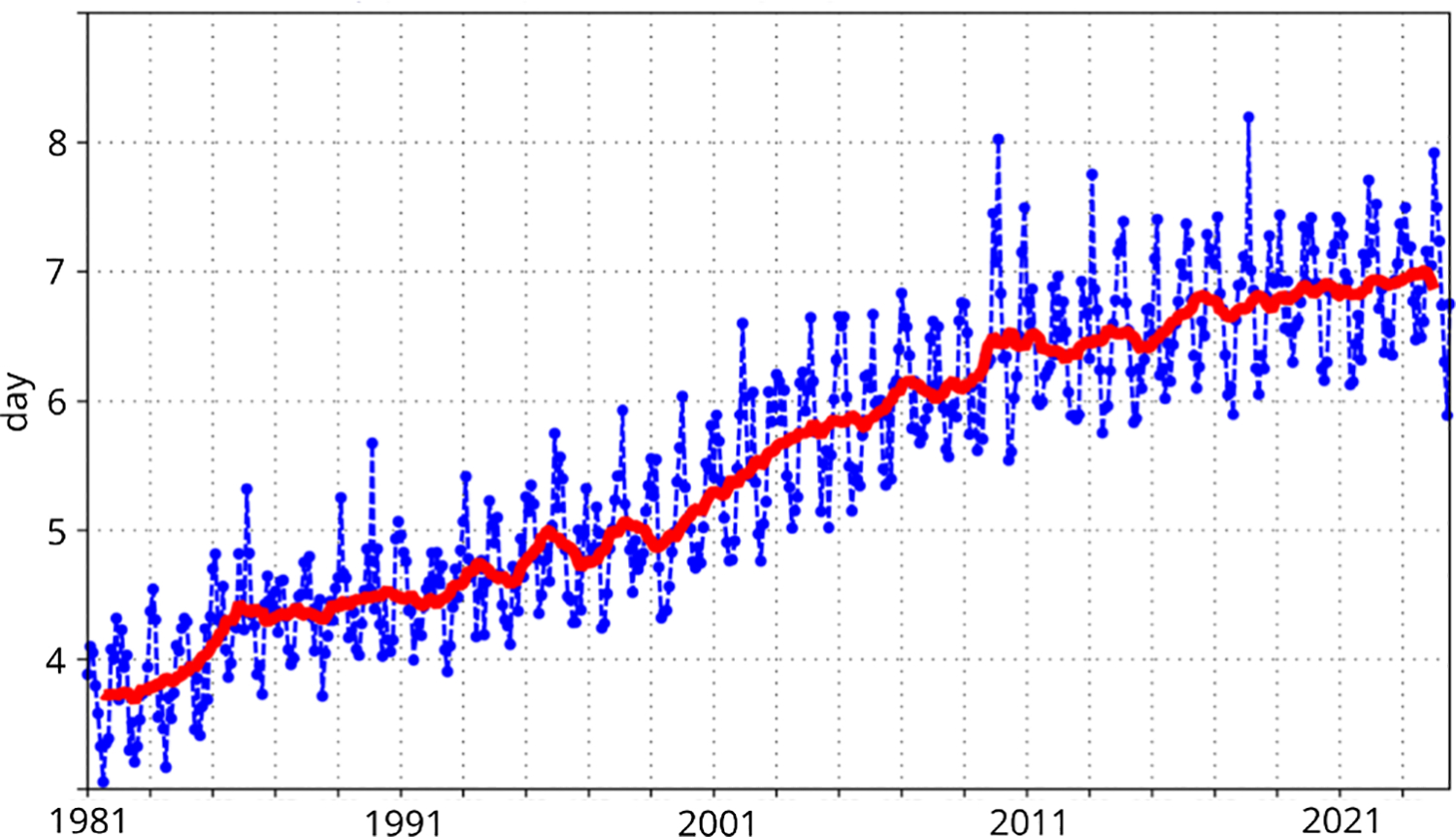
Evolution with time of the 500 hPa geopotential height forecast performance – each point on the curves is the forecast range at which the monthly mean (blue lines) or 12-month mean centered on that month (red line) of the forecast anomaly correlation (ACC) with the verifying analysis falls below 80% for northern hemisphere extratropics. *Figure from* Haiden et al. (2024) (CC-BY 4.0), *we have modified the labels from the original figure for better readability*.

In Figure 3 we reproduce one of the result that they report Haiden et al. (2024). This figure shows the lead time for a fixed Anomaly Correlation Coefficient of the 500hPA geopotential height forecast performance, that they are able to measure and how it evolved through time. Although this measure is similar to what we hope to capture through our grand social challenges, it asks some questions related to computing: How has the use of computing resource evolved throughout this evaluation? What is the gain related to new data collection, new models, and simply more compute time?

**Figure 4.**
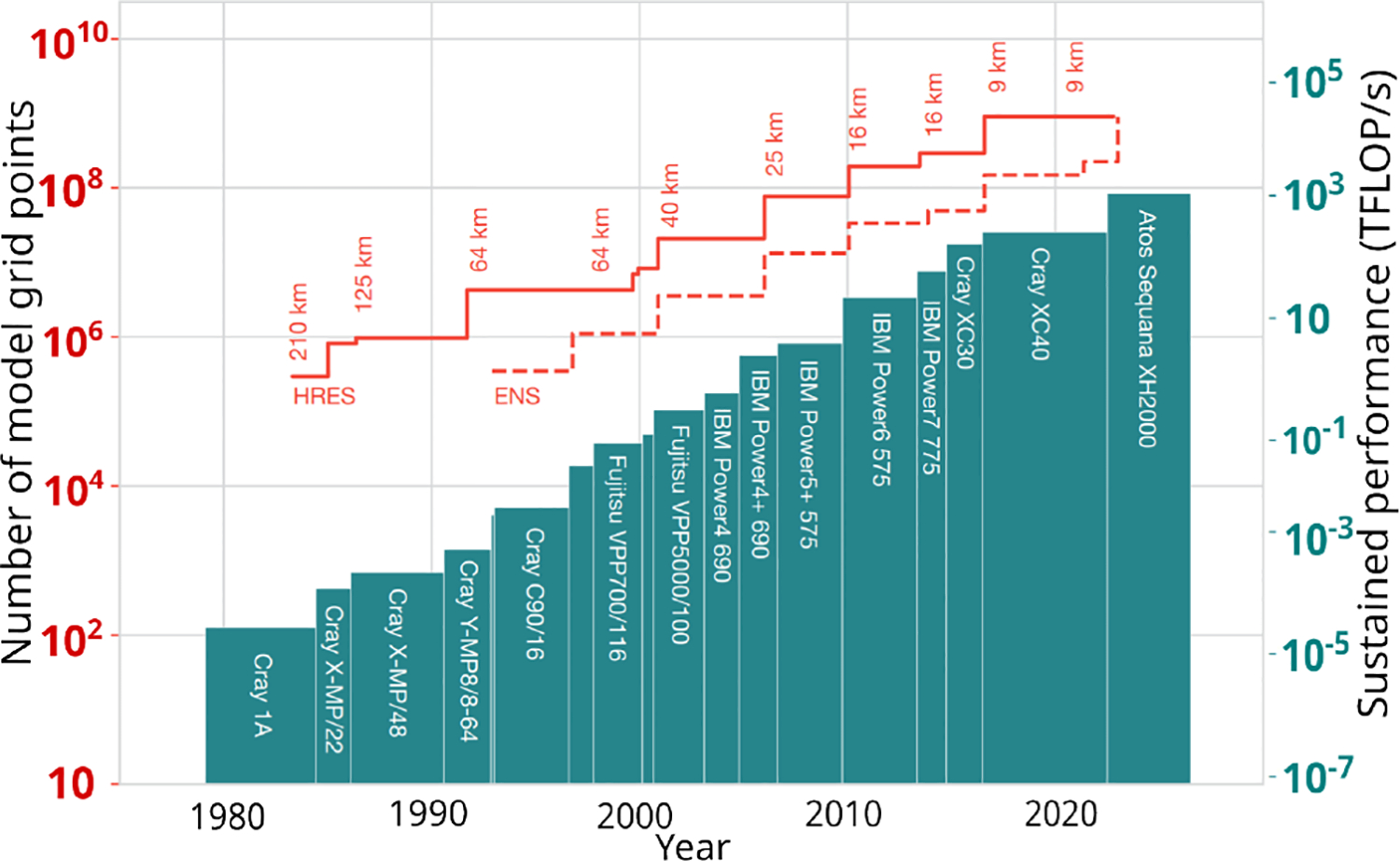
Chart showing the increase in model resolution and high-performance computing facilities used by ECMWF to compute High-Resolution forecasts (HRES) and Ensemble Forecasts (ENS). *Figure from* ECMWF (2024) (CC-BY 4.0), *we have modified the labels from the original figure for better readability*.

Some elements to these answers can be found in the 2023 ECMWF Annual report ECMWF (2024) which we reproduce in Figure 4.

A first rough observation from Figure 3 and Figure 4 is that between the year 2010 and 2024, the peak performance of the machine used by ECMWF has grown by roughly three orders of magnitude, while the lead time for anomaly detection at 80% precision has increased from roughly 6.5 days to 7 days.

Of course, the machine has different uses, and in this case we do not know whether a simulation uses the full scale of the machine, but all of these questions show why this problem is hard. In our case, we would be interested at a longitudinal study for a fixed point of time, how the performance evolves based on the compute resource used, to understand the scale needed for a given analysis.

A final note on this challenge: the challenge used here has been selected for illustration purpose. It would not have to be the selected challenge by the climate community, nor does it have to be the only challenge. In the next Sections we describe how a challenge can be selected.

#### Cancer therapy

A social challenge relevant to reducing cancer burden could be around the development of new therapies with fewer side effects.

Developing new cancer therapies with reduced side effects is crucial because current treatments, such as chemotherapy and radiation, often cause significant harm to healthy tissues alongside targeting cancer cells. These side effects can lead to severe complications, diminish patients’ quality of life, and limit the intensity or duration of treatment. By advancing therapies that are more precise and less toxic, we can improve patient outcomes, enhance quality of life, and reduce the physical and emotional burdens associated with cancer care.

Polypharmacological networks help us understand how drugs interact with all proteins in our bodies and are useful to develop drugs Ellingson et al. (2014); Funari et al. (2024). The polypharmacological, promiscuous nature of pharmaceuticals can have both beneficial and detrimental consequences. The former can be exploited to improve drug efficacy and prevent drug resistance by developing drugs that intentionally target multiple proteins in a disease pathway, and the latter can be used to avoid developing drugs with adverse reactions.

In this context, the social challenges becomes that of finding a set of drugs *A* of reasonnable size for real-life testing. The way to do this is to pose the problem in terms of the size of the drug-protein binding space one can explore, as well as the number of properties that can be tested. For a given disease, a particular pathway(s) is altered and contains a set of proteins, *X*. A set of proteins *Y* does not contain proteins from *X* and we want to limit the propensity to bind to these so that the drug has a higher specificity to the disease pathway. There is also a toxicity screen, *Z*, with proteins in which we want to develop drugs to not interact. *X*, *Y*, and *Z* are unique sets of protein conformers, and |*X|* + |*Y|* + |*Z|* is the total number of proteins in the large-scale screen which could be in the millions Aebersold et al. (2018), the number of unique protein conformers in a human. To find the set of drugs *A* for experimental testing, we want to find a set that for each *a* ∈ *A*,

$$S\left(a,x\right)>S\left(a,y\right)\gg S\left(a,z\right)$$

for as many *x* ∈ *X*, *y* ∈ *Y*, and *z* ∈ *Z* where *S* is the propensity of binding score between a drug and a protein and the greater the score the stronger the drug binds to the protein. *D* is the set of all drugs tested in the large-scale screen and the larger the size of *D*, the more likely you will find the optimal set *A*. The maximum size of *D* is of the order of 10^60^ Opassi et al. (2018), the estimated size of chemical space.

Hence in this case, the *weak-scaling* problem/scientific question would be to increase the size of *D*, the number of drugs tested, with the size of the machine while the *result-scaling*/social question is to measure how the size of *A* increases with the size of the machine used.

For this social challenge, it should be noted that in silico experiments do not necessarily provide real solutions to the actual challenge (finding a therapy). Another more complex measure could be to look at the success rate of potential drugs selected using these calculations vs the success rate of potential drugs selected without the additional calculations to understand the side effects. Success rates may be from various stages of the drug discovery and development pipeline, such as in cell-based assays, animal studies, or clinical trials.

#### Other examples of social challenges

For the sake of illustration we propose other examples with extremely simple descriptions:

- The consequences of an earthquake on a city as a function of the distance to its epicenter.
- Minimum time to analyze genomic sequences when needed for an urgent diagnosis and clinical decision (this is a case where strong scaling is a measure of the social challenge).
- Modeling X% of the brain’s response to a drug at a speed 100x faster than biological time.

Of course, all these questions need to be more accurately formalized as we are going to see in the next example.

### How do we select a Grand Social Challenge?

The selection of HPC Grand Social Challenges is a hard problem for many reasons:

#### Multidisciplinarity and Expertise:

There are a tremendous volume of applications running at HPC centers, some less known than others for instance Coral-reef analysis Tsai et al. (2019), digitalization of pinned insect collection Hereld and Ferrier (2019), or detection of racial gerrymandering Liu et al. (2016).

The selection of a grand challenge is a hard problem and needs expert review, both from the field, and experts with perspective from political sciences, economics and/or human sciences.

##### Proposed solution

To solve this issue, we propose that instead of having a single committee decide on challenges, the challenges are to be submitted by the community. Each submission would first be evaluated by domain-specific experts to approve the feasability of the grand challenge given the state of the art. Then as a second step, a steering committee including a variety of expertise from ethics, philosophy, political science and HPC would take a decision evaluating the social impact criteria.

Note that this may not be enough as even within a field, experts may disagree on the methodology to measure a social challenge. One of the best known example is brain simulation^§^where opposing approaches do not have the same answers to what constitutes a brain model.

However in this case, there is nothing that forbids the selection of both approaches as possible measures for the same social question, until the scientific community reaches a consensus.

#### Evaluation of the challenge, reproducibility:

Given that we are not evaluating a benchmark anymore but a social question, evaluation is much harder. Specifically, (i) there may be several pieces of software to perform the same type of evaluation; (ii) validating a result may need excessive resources/may not even be doable on another machine than the one it ran on.

Hence the question on how to trust a result is fundamental.

##### Proposed solution

Instead of re-running a software to compare the result, we propose to associate to each *Performance* result a reproducibility report detailing each version of the software used. This report should be evaluated by an expert committee to see whether the result is expected to be reproducible. Then we expect that if a result is particularly good, others will want to reproduce it (at their own scale) on their machines.

**Challenge selection process**

Based on the previous section, we propose the following process for challenge selection:

1. An individual or group of people submit a grand challenge;
2. This challenge is peer-reviewed by disciplinary experts;
3. It is selected by a multidiciplinary panel.

Because of the political implications around social challenges, all steps of this process should be transparent, which is possible through open peer-review.

### Challenge submission

In Table 1, we propose elements that should be included in a challenge submission form

One way to validate a challenge would be to have a list of applications that have been demonstrated to perform correctly on this problem. This also means that we need to be able to update this list.

In addition, while the initial Challenge submission form may not be complete, after acceptance, there should be a call to the community to update this list. Because there is a lot of politics involved (this may give a large visibility to some software), we may want transparency on decisions (i.e. open submissions and open decisions).

### Limits of Grand Social Challenges

There are many limits to producing such a list of challenges. Much consideration should be given to how the list may lead to negative consequences and efforts sould be made to ensure it facilitates advancements in the social challenges and smart decision making for the future of scientific computing.

#### Incompleteness

This grand challenge list cannot be used and should not be used to evaluate the social impact of an HPC center. As discussed earlier, there are a tremendous amount of HPC applications, it would be a mistake to think that this list describes fully what an HPC center does.

In addition, not all applications of HPC can be included in these Grand social Challenges:

- Many HPC applications do not have immediate social interests, HPC is an important factor of scientific discovery, i.e. the fundamental justification of science.
- Some of the advances allowed by HPC are event-based, and we cannot measure how the field improves, such as the discovery of Higgs-Boson particle in High-Energy Physics.

This incompleteness should not however prevent us from trying to evaluate some of the impact, in particular when trying to motivate whether a question should be ported at a much larger scale.

#### Simplicity

Even in fields that may be measured in terms of social advances and included in this list: the description and evaluation that we propose is a simple one. This is by design: the goal is to provide a first way to describe social advances allowed by HPC. However these scientific domains are obviously more complex. Particularly, some simulation results can have multiple uses.

## Conclusion

In this proposal, we argue that we do not evaluate enough the relation between the increasing scale of HPC machines, and the concrete social benefit obtained from their use. Yet, in a world where sobriety becomes critical to tackle the upcoming ecological disaster Kikstra et al. (2022), understanding why we do computations is fundamental. This is why we argue on the importance of evaluating *Result-Scalability*, essentially by showcasing the scalability of a computation through its output instead of through its input.

To make this community-change, we propose a multidisciplinary approach that allows us to measure in some domains how HPC allowed and continues to allow social advance. The measurement not only allows to show the gain permitted by architectural advances, but also the gain through new algorithmic design. This approach is another way to evaluate our computing infrastructure with a different objective: our responsibility toward society.

Like all metrics, this metric has some limits and risks. In particular, it should be extremely clear that these challenges do not permit to compare the social advance between fields, nor do they allow to measure the social impact of a given supercomputer. They are here to show, on specific social questions what scalability effectively brings.

Ultimately, showcasing the result scalability of an application should allow us to question the scale at which we run some applications.

## Figures and Tables

**Table 1.** Challenge submission form

Opt.?	Field	Description

	Grand challenge Description	A general description of the problems behind the grand challenge. It could be formulated as a question.
	Social Advance	Why does this contribute to social advancement? How is this used? This section should try to be as exhaustive as possible, not focusing solely on what could be seen as *positive* uses.
	Measure of social impact	How do we evaluate this challenge. This can be a runtime objective for time sensitive problems, or a scale perspective for modeling, prediction problems.
x	Secondary Objectives	For instance if the measurement is about the scale of the problem, this additional data could be the runtime to obtain the result.
	Validation	How can we evaluate the validity of the result?
	Existing software	What are existing software/application able to solve this problem?
x	Related Work	What are known performance for this objective? (+references)
x	Evaluation	Proposed name of reviewers/field experts

## Footnotes

*
https://supercomputing.org/

†
https://awards.acm.org/bell

‡
https://en.wikipedia.org/wiki/Nuremberg_Code

§
https://www.scientificamerican.com/article/massive-brain-simulators-seung-conntectome/
